# Microwave-Assisted Hydrothermal Synthesis of Pure-Phase Sodalite (>99 wt.%) in Suspension: Methodology Design and Verification

**DOI:** 10.3390/ma17010269

**Published:** 2024-01-04

**Authors:** Kamila Rouchalová, Dana Rouchalová, Vladimír Čablík, Dalibor Matýsek

**Affiliations:** 1 Department of Environmental Engineering, Faculty of Mining and Geology, VŠB—Technical University of Ostrava, 17 Listopadu 2172/15, 708 00 Ostrava-Poruba, Czech Republic; dana.rouchalova@vsb.cz (D.R.); vladimir.cablik@vsb.cz (V.Č.); 2 Department of Geological Engineering, Faculty of Mining and Geology, VŠB—Technical University of Ostrava, 17 Listopadu 2172/15, 708 00 Ostrava-Poruba, Czech Republic; dalibor.matysek@vsb.cz

**Keywords:** coal fly ash, zeolite, hydrothermal synthesis, microwave irradiation, single phase, sodalite, XRD

## Abstract

Despite numerous studies focused on the hydrothermal (HT) synthesis of fly ash zeolites (FAZs), this method still has many limitations, the main of which is the low yield of zeolites. Hydrothermally synthesized zeolites are typically multiphase and exhibit low purity, which limits their applicability. Pure-phase zeolites have been primarily prepared from filtrates after alkaline mineralization of fly ashes, not directly in suspension. In addition, the published methodologies have not been tested in a wider set of samples, and thus their reproducibility is not confirmed. The aim of the study is to propose a reproducible methodology that overcomes the mentioned limitations. The influence of the Si/Al ratio (1.3:1–1:2), the type and concentration of the activator (2/4 M NaOH/KOH/LiOH), the reagent (30% LiCl), the duration (24–168 h), and the temperature (50–180 °C) of the synthesis phases were studied. The sequence of the synthesis phases was also optimized, depending on the type of heat transfer. The fly ashes were analyzed by wavelength-dispersive X-ray fluorescence (WD XRF), flame atomic absorption spectrometry (F-AAS), and X-ray diffraction (XRD). The energy intensity of the synthesis was reduced through the application of unique microwave digestion technology. Both microwave and combined (microwave and convection) syntheses were conducted. FAZs were identified and quantified by XRD analysis. This study presents a three-stage (TS) hydrothermal synthesis of pure-phase sodalite in suspension. Sodalite (>99 wt.%) was prepared from nine fly ashes under the following conditions: I. microwave phase: 120 °C, 150 min, solid-to-liquid ratio (S/L) 1:5, Si/Al ratio 1:1.5, and 4 M NaOH; II. convection phase: 120 °C, 24 h, S/L 1:40, and the addition of 30 mL of 30% LiCl; and III. crystallization: 70 °C for 24 h. The formation of rhombododecahedral sodalite crystals was confirmed by scanning electron microscope (SEM) images.

## 1. Introduction

If fly ashes do not comply with legislative and normative regulations, they are often used as foundations in infrastructure construction and backfilling or stored in a landfill. In the case of higher concentrations of toxic substances, fly ashes may pose a risk of leakage. The synthesis of FAZs and their standardized utilization in construction (additives and binders) aim to utilize the elemental potential of fly ashes. For numerous applications, the demand for zeolites exceeds the resources of their natural counterparts. However, studies of the synthesis of FAZs arrive at different results, and even the mechanism of the zeolitization of FAZs has not been fully clarified. The design of a reproducible methodology is limited by the variable elemental and mineral composition of fly ashes.

In one-step convection syntheses, the content of FAZs in the synthesis product reached 2.09–65 wt.% [1,2,3,4,5,6,7,8,9,10,11,12]. The disadvantage of lower contents is the need to separate the zeolites from the product. Although the quantification of FAZs is lacking in many studies, highly crystalline products (but not >99 wt.%) were prepared in several syntheses due to high temperatures or long treatment times. Two-step convective syntheses typically involve activation (intense stirring) and long-term high-temperature convective heating (without stirring) [13,14]. Maingayne et al. [13], after the activation of fly ash in 5 M NaOH (48 h, 47 °C, S/L 1:5, agitation speed 200 rpm) and subsequent 48 h of heating at 140 °C (S/L 1:12.5), prepared zeolite Na-P1 (94.7% crystallinity). Under analogous conditions, Musyoka et al. [14] prepared highly pure zeolite Na-P1 [14]. In a low-temperature but long-term two-stage synthesis (dissolution: 4 h, 104 °C, 2 M NaOH, S/L ratio 1:200; convection 96 h, 80 °C), Wałek et al. [15] prepared a product with up to 80 wt.% of zeolite Na-P1. However, even extended reaction times and higher temperatures may not lead to the synthesis of products with a high crystallinity of FAZs. After activating fly ash in 2 M NaOH at room temperature (6 days) and convection heating (24 h, 150 °C), Manique et al. [16] synthesized sodalite with a large amount of unreacted quartz and mullite.

High-purity zeolites (>95 wt.%) were synthesized from the filtrate after the alkaline activation of fly ash. The extract was enriched with aluminum and left to crystallize for 48 h at 90 °C. From 1 kg of fly ash, 50–85 g of zeolite was obtained [1]. Highly pure FAZs were prepared using a two-stage convective extraction method in other studies as well [17,18,19,20]. However, the aim of this study is to propose a procedurally simpler methodology for the synthesis of FAZ in suspension. The advantage of the synthesis of FAZs directly in suspension is that there is no production of solid-phase waste from extract preparation. Thirteen different zeolites were prepared from one fly ash by modifying the reaction parameters [11]. This study is focused on the synthesis of a specific zeolitic phase, i.e., sodalite. Wałek et al. [15] prepared in the NaOH concentration range of 0.5–4 mol∙L^−1^ the largest amount of hydroxysodalite after 96 h of convection synthesis at 104 °C using 4 M NaOH. Querol et al. [11] determined the following optimal conditions for the convection synthesis of hydroxysodalite: 5 M NaOH, S/L 1:18, and temperature 150–200 °C. Fukasawa et al. [21] promoted the nucleation of hydroxysodalite crystals by pulverization. The crystallinity of sodalite in the product can also be increased as a result of the phase transformation of initially formed zeotypes into more stable forms (Ostwald’s rule) [13]. Zeolite framework types LTA and FAU can be transformed to sodalite by prolonging the time of convection [22] or microwave heating [23]. The transformation of the GIS type (zeolite Na-P1) to hydroxysodalite due to an increased stirring rate [13] or higher activator concentration [5] was also observed.

Zeolites were mostly prepared only from fly ash, an alkaline solution, or an aluminum source [4,5,6,7,8,9,10,11,12,13,15,16,17,18,19,20,21,23,24,25,26,27,28,29,30,31,32,33,34,35,36,37]. Activators such as NaOH [4,6,7,8,9,10,11,12,13,15,17,18,19,20,21,23,24,25,26,27,28,29,30,31,32,33,34,35,37], KOH [5,6,8,11,35,36,38], and Na_2_CO_3_ were investigated [5]. Zeolites were also synthesized in a mixture of two solutions, NaOH and KOH [8], or NaOH/KOH and Na_2_CO_3_ [5]. However, studies on the influence of reagents on the crystallization of a specific type of zeolite are lacking. Kunecki et al. [39] added 3 M NaCl to the mixture to synthesize sodalite. Fukui et al. [40] detected hydroxysodalite and phillipsite in the product of microwave synthesis in suspension (9 h, 100 °C, 2 M NaOH, S/L was 1:25) after the addition of 1.5 M NaCl. However, all the quartz from the fly ash was unreacted. In this study, the effect of LiCl was studied.

FAZs were prepared by microwave synthesis in suspension [21,24,30,34,40] and from extract [23,25,27,32,33]. Methodologies with combined heating (microwave and convection) were also proposed [23,25,26,31,32,41]. Despite the combined heating, the yields of zeolites were not high. Bukhari et al. [31] achieved a yield of FAZs of 32–37%. A product with a higher zeolite content was prepared if microwave irradiation was preceded by convection treatment (12 h at 60 °C) rather than microwave melting (2 h, 550 °C). Behin et al. [41] prepared a product with a FAZ crystallinity of up to 67.24% by combining convection synthesis and subsequent 30 min of microwave irradiation (300 W) in a microwave oven. Pure-phase sodalite was prepared from the microwave extract, which was subsequently irradiated with ultrasound (20 min, 600 W) [25] or conventionally heated for 1 h in a drying oven [23]. However, when FAZs were synthesized in suspension, the product contained sodalite with a large amount of ash residues, mainly mullite [25]. It remains unanswered if it is possible to prepare pure-phase sodalite by combined heating (microwave and convection) in suspension. This study complements the HT synthesis of FAZs with the unique microwave digestion technology of an SRC (Single Reaction Chamber). Unlike many methodologies [21,23,24,25,26,27,31,32,33,34,41], the synthesis mixtures in the UltraCLAVE IV autoclave were subjected not only to radiation but also increasing pressure. The application of microwave irradiation makes our methodology economically acceptable. The times and temperatures of the individual synthesis phases did not exceed 24 h and 120 °C. This study presents a reproducible methodology that integrates not only determined reaction optima but also the sequence of synthesis steps depending on the type of heat transfer.

## 2. Materials and Methods

### 2.1. Tested Fly Ashes

Fly ashes are products of high-temperature combustion in granulation boilers (ČEZ, a. s., Praha, Czech Republic). Different fly ashes were used for the synthesis (Table 1).

### 2.2. Conducted Analyses and Determinations

The particle size distribution (PSD) was measured with a multilaser analyzer type 1190 (CILAS, Orléans, France). Granulometry was measured under the following conditions: mode: liquid dispersion, measurement range: 0.04–2500 µm, and dispersion medium: ethanol. The precision and reproducibility of the measurements were <3% and <1%, respectively.

Analytical crystal LiF220 was used. The major elements were evaluated by the QUANT EXPRESS module (standard-free) in the form of stable oxides (SiO_2_, Al_2_O_3_, CaO, Fe_2_O_3_, K_2_O, TiO_2_, MgO, Na_2_O, MnO, P_2_O_5_, and SO_3_) in wt.%. Elemental contents were recalculated. The minor elements were evaluated by the GEO-QUANT T calibration module in elemental form in ppm concentration. Measurement uncertainties for major and minor elements are 10% and 20%, respectively. The measurement uncertainty for the major and minor elements is 10% and 20%, respectively.

The pH values of fly ash leachates were determined according to Marrero et al. [42]. The leachate was prepared in a ratio of 1:2.5 (*w*/*v*). The suspension was shaken for 60 min on a Standard 1000 orbital shaker (VWR International, Radnor, PA, USA). After standing for 60 min, the pH value was measured with a pH meter 330i/Set (WTW, Weilheim, Germany) at 25.4 °C.

Loss on ignition (LOI) was determined according to the ČSN 72 0103 standard. The sample (1 g) was repeatedly heated at 1100 °C (1 h) in a mikroTHERM 600 muffle furnace (LAC, s.r.o., Židlochovice, Czech Republic) until the weight stabilized. The LOI was determined three times for each sample. For an LOI in the range of 1–5%, the trueness and precision of the determination are 0.16% and 0.25%, respectively. For an LOI in the range of 5–10%, the trueness and precision of the determination are 0.28% and 0.39%, respectively [43].

The concentrations of Al and Fe in the fly ashes were determined using a Thermo Elemental SOLAAR M atomic absorption spectrometer (Analytical Technology, Inc. UNICAM, Delph, UK). The samples were dissolved in a mixture of HF, HCl, and HNO_3_. Iron concentrations in fractions after magnetic separation were determined by F-AAS. Data from AAS were used for the calculations of technological indicators of magnetic unbundling.

Mineralogical analysis was determined using an Advance D8 X-ray diffractometer (BRUKER AXS GmbH, Rheinstetten, Germany). The samples were measured in the Bruker AXS Diffrac (version 2) software. Data were evaluated using the Bruker AXS Eva (qualitative) and Bruker AXS Topas (quantitative) modules. The measurement parameters were 25 °C, an initial detector angle of 5°, a step size of 0.04°, and a final angular position of 2θ 8°.

The microstructure was studied with a Quanta 650 FEG scanning electron microscope (Thermo Fisher Scientific, Waltham, MA, USA). An Octane Elect detector (EDAX, Mahwah, NJ, USA) was used for spot ED XRF analysis.

### 2.3. Pretreatment of Samples

The fly ashes were ground using a VMA—386 vibratory mill (VIPO, Czechoslovakia). Ground samples were sieved through stainless steel analytical sieves (pore sizes 100 µm), while the sub-sieve fraction was used for syntheses. The grinding of fly ash was also carried out in other methodologies [39,44]. Wałek et al. [15] determined that particle size reduction can accelerate the dissolution of fly ash in an alkaline activator.

#### Magnetic Separation

Iron already inhibits FAZ nucleation at very low concentrations [36,45]. In many methodologies, the iron content (2.31–12.24%) was not reduced [3,6,10,12,13,16,19,24,25,29,32,34,36,46]. Iron concentration was reduced by a high-gradient magnetic separator [7] or leaching in HCl [44,47]. Leaching in HCl reduced the iron content, quantified as Fe_2_O_3_, from 5.53% to 0.03% [47]. According to Vassilev et al. [48], the efficiency of dry separation was tested using a magnetic drum separator (Mechanical Workshops Prague, Czechoslovakia). The magnetic induction intensity was 0.8 T. However, due to the separation of approx. 87 wt.% of the sample into the magnetic fraction, the induction roller separator for fly ash separation was evaluated as unsuitable. Vassilev et al. [48] successfully separated the magnetic fraction of fly ash using a drum magnetic separator. The yield of the magnetic concentrate was in the range of 0.7–4.1%. The magnetic concentrate mainly contained magnetic minerals and oxides of Fe, Mg, Ti, Mn, and Cr. Finally, in this study, magnetic separation was performed using a low-intensity Davis magnetic tube concentrator (Czechoslovakia).

Magnetic separation was evaluated by the following parameters: yield, recovery, and efficiency [49].

Yields were calculated using the following equations:

(1) $$v_{c}=\frac{a-b}{c-b}\times 100$$

(2) $$v_{b}=\frac{c-a}{c-b}\times 100$$

In these equations, *v_c_* represents the yield of magnetic concentrate (%); *v_b_* represents the yield of non-magnetic waste (%); *a* is the iron content in fly ash (%); *b* is the iron content in non-magnetic waste (%); and *c* represents the iron content in the magnetic concentrate (%).

Recoveries were calculated using the following equations:

(3) $$m_{c}=\frac{C_{r}}{A_{r}}\times 100$$

(4) $$m_{b}=\frac{B_{r}}{A_{r}}\times 100$$

(5) $$w_{c}=\frac{C_{b}}{A_{b}}\times 100$$

(6) $$w_{b}=\frac{B_{b}}{A_{b}}\times 100$$

(7) $$m_{c}+m_{b}=100$$

(8) $$w_{c}+w_{b}=100$$

(9) $$C_{r}=v_{c}\times c$$

(10) $$B_{r}=v_{b}\times b$$

(11) $$A_{r}=100\times a$$

(12) $$C_{b}=v_{c}\times c_{b}$$

(13) $$B_{b}=v_{b}\times {\;b}_{b}$$

(14) $$A_{b}=100\times a_{b}$$

In these formulas, *m_c_* represents the metal recovery to the concentrate (%); *m_b_* represents the metal recovery to the waste (%); *w_c_* is the gangue recovery to the concentrate (%); *w_b_* is the gangue recovery to the waste (%); *C_r_* represents the metal content in the concentrate (%); *B_r_* is the metal content in the waste (%); *A_r_* is the metal content in the fly ash (%); *C_b_* represents the gangue content in the concentrate (%); *B_b_* symbolizes the gangue content in the waste (%); and *A_b_* symbolizes the gangue content in the feed (%).

Efficiencies were calculated using the following equations:

(15) $$\eta_{c}=m_{c}\times w_{c}$$

(16) $$\eta_{b}=m_{b}\times w_{b}$$

(17) $$\eta_{c}=-\eta_{b}$$

In the formulas, *η_c_* symbolizes the technological efficiency relative to the concentrate (%) and *η_b_* symbolizes the technological efficiency relative to the waste (%).

### 2.4. Hydrothermal Synthesis of FAZs

Compared to convective heating, microwave irradiation accelerates the dissolution of Si^4+^ and Al^3+^ from fly ash [23,24,25,27,33], and heating occurs throughout the entire volume [24,27]. Methodologies were proposed in which microwave radiation was included before [24,25] and behind the conventional stage [26,27,31,41]. Inada et al. [24] observed a positive effect of microwaves during the synthesis of FAZs in the initial (dissolution) but not the middle and late phases of zeolitization. The formation of FAZs in the final stage is the result of a cooperation of the dissolution of the aluminosilicate gel, which is the product of the second stage of zeolitization and re-precipitation. Microwave radiation accelerates the dissolution of aluminosilicate gel. However, a secondary consequence of microwave radiation is the formation of active water molecules, which are created by breaking the intermolecular hydrogen bridges of an aqueous alkaline solution [24]. These active molecules directly attack Si–O and Al–O bonds, cause the disintegration of unstable zeolitic nuclei, and thus inhibit their nucleation [28,31]. Microwave radiation can also reduce the rate of growth of already-formed zeolitic crystals [24,27]. In accordance with the mentioned studies, microwave radiation was included in the first stage of the TS synthesis. The duration of the synthesis phases were chosen according to the cited studies [4,6,7,8,9,10,11,12,13,15,19,21,24,25,26,27,28,29,30,31,32,33,34,37,38,50,51]. The zeolitization of fly ashes with a high content of the amorphous component was already recorded in a short time, i.e., 6–8 h [29,51]. However, achieving similar FAZ yields for fly ashes with a high quartz or mullite content required extending the alkaline activation time up to 24–48 h [51]. Querol et al. [38] determined that the size of FAZ crystals after a shorter activation time is 10–50 μm, while with increasing time, the dimensions of zeolitic aggregates can reach up to 300 μm. Itskos et al. [4] also confirmed that increasing the alkaline treatment time can lead to further crystal growth.

#### 2.4.1. I. Phase—Microwave Digestion

**Figure 1:**
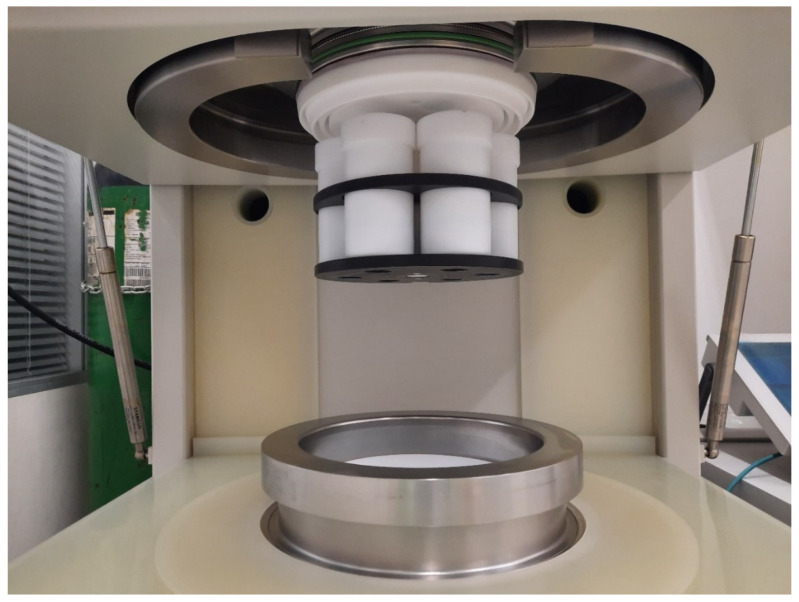
Reaction vessels in a six-position Weflon-PTFE holder mounted in an UltraCLAVE IV reactor.

Microwave digestion was performed in a pressure reactor in an UltraCLAVE IV autoclave (Milestone S.r.l., Sorisole, Italy). A 3.5 L PTFE insert with an absorption medium (300 mL 1.167 M NaOH) was put into the reactor. Microwave digestion was performed in 120 mL PTFE vessels placed in a six-position sample holder (Figure 1). The S/L ratio was 1:5 (5 g of fly ash and 25 mL of 2/4 M NaOH/KOH/LiOH). The selected activator was always used in all stages of synthesis. Depending on the Si/Al ratio (1.3:1–1:2), Al_2_O_3_ was added. In other studies, aluminum concentrations in reaction mixtures were increased using Al_2_O_3_ [3], NaAlO_2_ [16,17,18,23,25,27,31,33,41], aluminum foils [32,39], or waste solution from aluminum anodizing [19]. The mixtures were stirred automatically using cross-shaped magnetic stirrers at an intensity of 70%. After the preparation of the mixture, the sample vessels were closed with PTFE covers. The load pressure was 30 bar. The pressure was loaded manually directly from the gas bottle (N_2_) before starting the program. In high-temperature programs, the initial pressure should not be too high because the pressure could quickly reach the limit value. The maximum power was 1200 W. In the program, limit parameters were preset, and if exceeded, the radiation was interrupted. The maximum external temperature of the reactor was 80 °C, and the limit pressure was 130 bar. In the event of long-term alkaline decomposition of fly ash (>2 h), *T*_2_ was the limiting factor, which was set at the maximum possible value of 80 °C. The maximum measured pressure was 57.5 bar. Cooling was initiated above 30 °C (corresponding to *T*_2_). Decomposition temperatures were 120–180 °C. The total radiation time was 150 min, while the first 30 min always required heating to the set temperature. The pressure release temperature and the rate after the end of radiation were below 80 °C and 10 bar∙min^−1^, respectively.

#### 2.4.2. II. Phase—Convection Heating

Heating was conducted in 500 mL autoclavable PTFE bottles in a UF 110PLUS laboratory oven (Memmert GmbH + Co.KG, Schwabach, Germany) at a temperature of 120–180 °C. The heating time was 24 or 48 h. The S/L ratio was 1:40. A total of 30 mL of 30% LiCl was added to the mixture. Suspensions were mixed manually.

#### 2.4.3. III. Phase—Crystallization

The inclusion of crystallization was one of the factors that using the methodology of Längauer et al. [3] led to an increase in the crystallinity of FAZs. The mixtures were heated (1 or 7 days) at 50 °C or 70 °C in 500 mL PTFE bottles in a Memmert UF 110PLUS drying oven. The mixtures were not stirred.

**Scheme 1:**
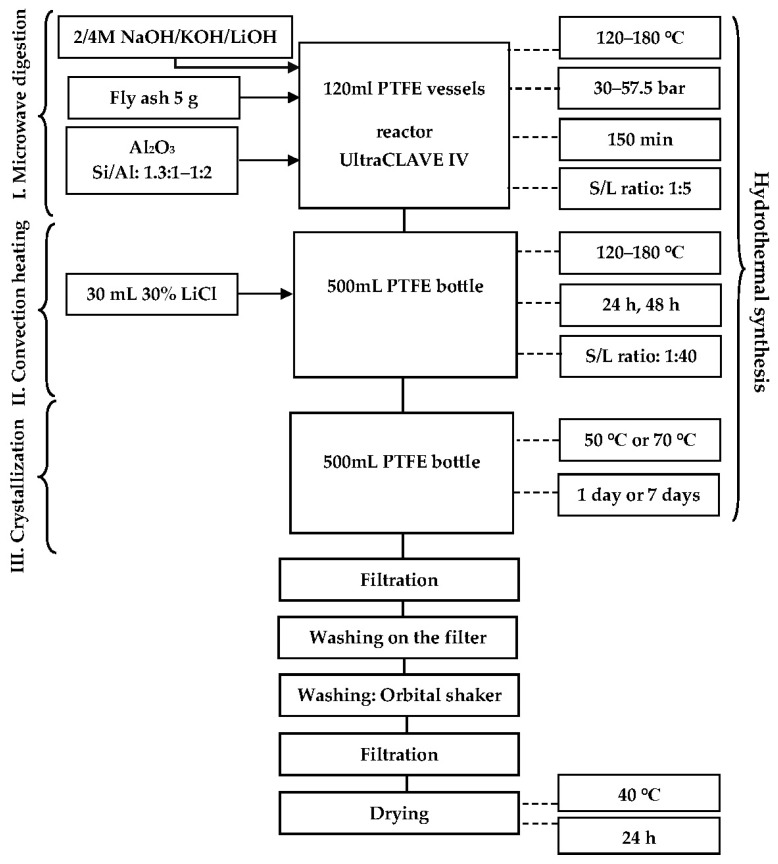
Three-stage HT synthesis of FAZs.

The solid phase was filtered and washed with 3 L of distilled water. The products (+150 mL of distilled water) were shaken for 30 min on a Standard 1000 orbital shaker (VWR International, Radnor, PA, USA) and filtered and dried for 24 h at 40 °C. Two types of experiments were performed: microwave synthesis (only Phase I) and three-stage synthesis. The scheme of the TS synthesis is shown in Scheme 1.

Furthermore, experiments VL_20 and E183 were conducted. In the three-phase hydrothermal synthesis of VL_20, microwave decomposition was carried out directly in a 3.5 L PTFE insert, leading to an increase in the amount of decomposed sample to 50 g. In experiment VL_20, sample No. 1 LED was used. The other parameters of the VL_20 synthesis were equal to the determined optimal conditions. To compare the efficiency of long-term HT synthesis with minimal energy consumption, experiment E183 was conducted. The convective one-step synthesis of E183 was performed in a 500 mL PTFE bottle. Synthesis parameters were 4 M NaOH, 30 mL 30% LiCl addition, S/L 1:40, and Si/Al ratio 1:1.5 (determined optimal conditions of the II. Phase). In experiment E183, sample No. 1 LED was used. The mixture was left for 183 days at 20 ± 2 °C.

## 3. Results and Discussion

### 3.1. Characterization of the Fly Ashes

**Figure 2:**
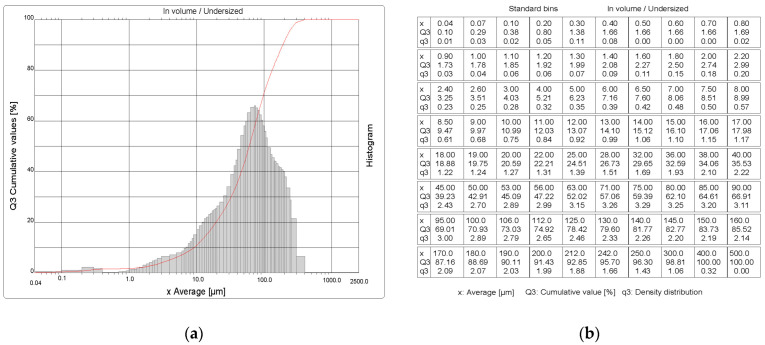
Particle size distribution of the LED sample: (**a**) histogram and cumulative sum curve; (**b**) quantification of the limit values of the size intervals x, their frequency q3, and the cumulative values Q.

The particle size distribution indicates that the LED sample is 99.9% made up of particles with a size of 0.04–400 µm (Figure 2). From the cumulative distribution curve, it was read that the particle diameter at 10% is 9.03 µm, the diameter at 50% is 59.95 µm, the diameter at 90% is 189.21 µm, and the average particle diameter is 80.86 µm.

FAZs were synthesized from fly ashes of variable chemical compositions (Table 2 and Table 3).

Vassilev and Vassileva [52] determined 13.32–27.91% Si and 6.62–18.84% Al in 41 bituminous and lignite fly ashes. Tested fly ashes contain at least 21.36% Si and 11.15% Al (Table 2), and are thus above-average enriched in essential zeolitic elements. The tested ashes contain at least 21.36% Si and 11.15% Al and are, therefore, above-average enriched with basic zeolitic elements. It is clear from the results of the XRF analysis that bituminous fly ashes do not have a more optimal elemental composition than brown coal ashes for synthesis. The black coal samples (DET_E and DET_M) contain the least Al and the most Ca, which are a inhibitors during zeolitization [4]. However, other inhibitory elements such as Fe, Zn, Cu, Pb, Ni, As, Rb, and Cs were also detected in fly ashes [45]. Ca is usually contained in fly ashes as anhydrite, gypsum, or calcite, and Fe is mostly contained as hematite or magnetite [4]. The characteristics of the ashes were supplemented by determinations of the AAS (Fe, Al), LOI, and pH of the leachates (Table 4).

Metal contents determined by AAS (Table 4) were mostly lower than those analyzed by XRF. This could be due to the incomplete dissolution of samples in acids before AAS determination. Fly ashes are not easily soluble, even in aggressive mineralizers, and from the point of view of mineralogical composition, it is not clear which of their components are easier to zeolitize. FAZs were synthesized predominantly from the amorphous phase [4,8,24] but also from quartz and mullite [3]. Itskos et al. [4] prepared FAZs (24 h, 90 °C, 1 M NaOH) from the glass phase and muscovite (KAl_2_(AlSi_3_O_10_)(F,OH)_2_). It was deduced that the time required for zeolitization is inversely proportional to the amount of amorphous phase. The rate of nucleation of zeolitic crystals decreases the undissolved mullite and the unreacted glass phase [36,45,53]. The studied fly ashes exhibit variable mineralogical composition (Table 5).

**Figure 3:**
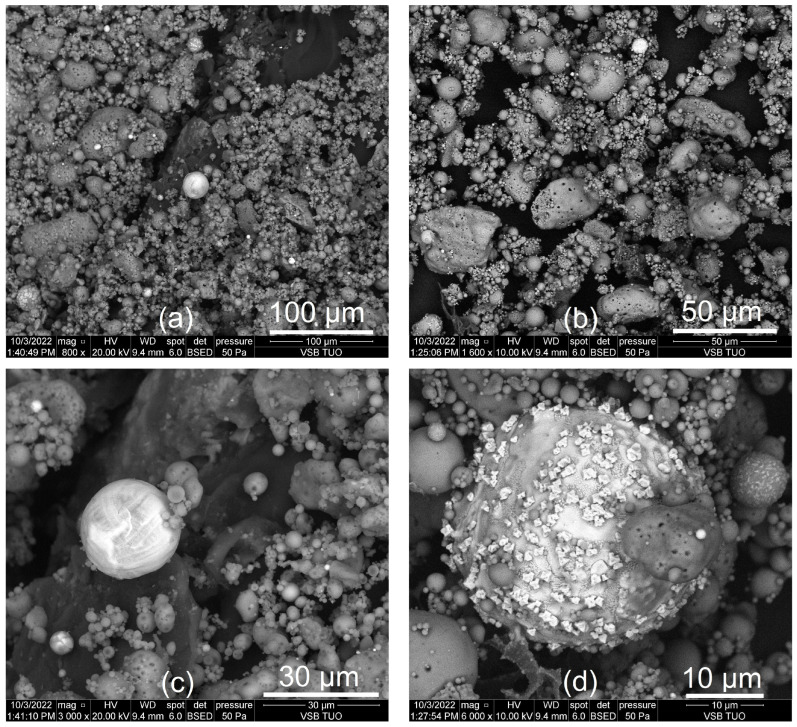
(**a**–**d**) SEM images of LED fly ash.

A higher quartz content (>9%) was determined in fly ashes from mechanical separators (TRM_M, DET_M) or samples with their share (MEL_II and MEL_III). Fly ash from the Mělník power plant, LED, and TRM_E samples also contains >31% mullite. Convective heating is effective for dissolving quartz and mullite [3], while microwaves decompose the glass phase [24,27]. Since fly ashes contain 50.54–81.76% of the amorphous phase, microwave digestion was included in the HT synthesis. However, the content of the glass phase was also increased by the unburned coal, which is also amorphous. The surface morphology of the LED fly ash was studied by SEM (Figure 3).

**Figure 4:**
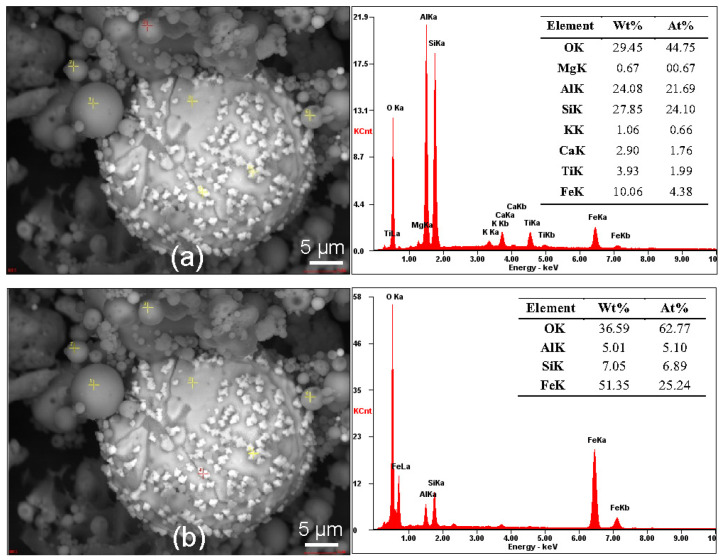
SEM image of LED sample supplemented with ED XRF spot analyses of the (red cross): (**a**) cenosphere; (**b**) microparticles on the microsphere surface.

In Figure 3c,d, clusters of spherical and allotriomorphic, i.e., completely limited in terms of idiomorphy, particles can be seen [54,55]. The spherical microsphere in Figure 3d has a radius of approx. 20 μm and its surface is covered with particles created, for example, by condensation. These microparticles are often composed of chlorides or sulfates of alkali metals and are a reservoir of elements such as As, Cd, Se, Be, B, and Fe. The size of fly ash microspheres is usually 20–200 μm [54]. Fly ash microspheres include cenospheres, whose cavities are usually filled with air, nitrogen, or carbon dioxide, and plerospheres, which are filled with smaller spherical particles. The cenospheres are mainly composed of O, Si, and Al at the atomic level [56], which is confirmed by the SEM image with ED XRF analysis (Figure 4a).

The results of SEM-ED XRF analysis (Figure 4) complement and confirm the results of XRD and WD XRF. Fly ashes are mainly composed of aluminosilicate glass, mullite, and quartz, i.e., amorphous and crystalline forms of SiO_2_ and Al_2_O_3_. The cenosphere is also composed of oxide forms of Fe (Fe_2_O_3_ and FeO), Ti, Mg, Ca, and K, which is also confirmed by Zanjad et al. [57]. SEM-ED XRF analysis (Figure 4b) confirms that the microparticles on the surface of the microspheres are a reservoir of heavy metals, such as Fe.

### 3.2. Magnetic Separation

The values of the magnetic separation indicators are shown in Table 6.

### 3.3. Synthesis Products

Different phases were analyzed by XRD in the HT synthesis products (Table 7). Zeolitic phases are marked in bold.

#### 3.3.1. Microwave Syntheses

**Scheme 2:**
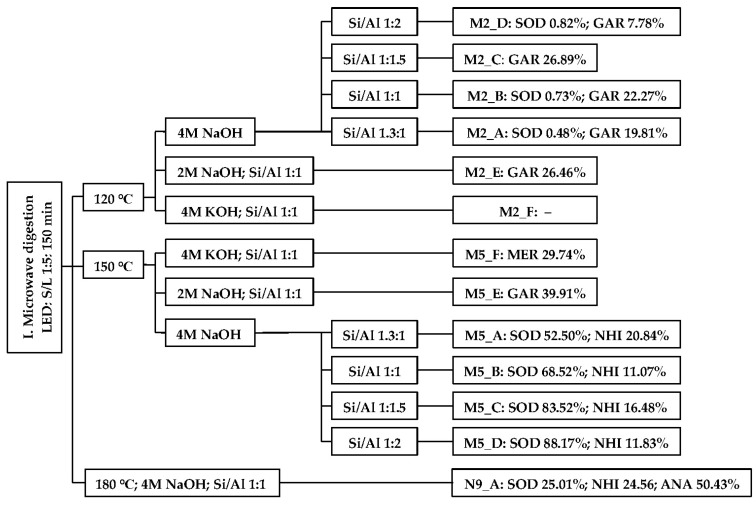
FAZs determined by XRD in microwave products depend on temperature, concentration and type of activator, and Si/Al ratio.

Sample No. 1 LED was used. Only microwave radiation (I. Phase) was performed. The types and content of FAZs in the products are a function of the synthesis parameters (Scheme 2).

After just 150 min of microwave irradiation at 120 °C (4 M NaOH, Si/Al 1.3:1—unmodified), 20.29% of FAZs were analyzed in the product M2_A. Matlob et al. [32] determined that the influence of parameters on the microwave dissolution of fly ashes in NaOH decreases in the following order: time, activator concentration, and power. The initial heating temperature was chosen in accordance with a study by Adamczyk and Białecka [29], who determined 120 °C as the lowest temperature at which FAZs were formed during HT synthesis in an autoclave. In the temperature range of 90–320 °C, the authors also established a positive correlation between the yield of FAZs and temperature.

At 120 °C (4 M NaOH) in the interval of Si/Al ratios 1.3:1–1:1.5 (inclusive), a positive correlation was found between aluminum concentration and FAZs yield. However, when the aluminum content was further increased (Si/Al 1:2), the yield of FAZs decreased (M2_D). At 150 °C (4 M NaOH) in the Si/Al interval 1.3:1–1:2, the sodalite content of the product was positively correlated with the aluminum content of the mixture. Increasing the aluminum concentration caused, at the expense of nepheline hydrate I (NHI), a gradual increase in sodalite crystallinity. A natural analog of NHI is the mineral fabriesite (Na_3_Al_3_Si_3_O_12_·2H_2_O), which was described at the jadeite deposit by Tawmaw-Hpakant [62]. Fabriesite represents a bond between zeolites and tectosilicates, which do not bind water in their structures. The fabriesite lattice contains channels that are occupied by sodium cations and water [63]. NHI was previously prepared at 200 °C from sodium aluminosilicate glass [64] or kaolinite [61] after NaOH activation. Synthetic NHI exhibits high HT stability. At 600 °C, reverse dehydration but not the destruction of the NHI aluminosilicate lattice was observed [62]. Garronite-Ca was prepared at 150 °C only in 2 M NaOH (M5_E), and its synthesis is thus conditioned by a lower concentration of NaOH or a lower temperature (M2_A–C). Therefore, a specific type of FAZ can be prepared only by changing the concentration or the type of activator. Under otherwise identical conditions (150 °C, Si/Al 1:1), sodalite mixed with NHI, garronite-Ca, and merlinoite were synthesized with 4 M NaOH (M5_A), 2 M NaOH (M5_E), or 4 M KOH (M5_F). The higher efficiency of microwave than convection heating can be seen by comparison with the study by Pedrolo et al. [36]. Pedrolo et al. [36] convectively synthesized a small amount of merlinoite at 100 °C and 150 °C for up to 72 h and 8 h, respectively.

Consistent with the study conducted by Duxson et al. [65], a higher activation effect of NaOH compared to KOH was confirmed. This could be attributed to the smaller size and easier mobility of Na+ in the hydrated gel network [65]. While hydroxyl ions act as catalysts for the release of Si^4+^ and Al^3+^ from fly ash, alkali metal ions balance the negative charge of the emerging tetrahedral coordination of aluminum [5,65]. Sodalite crystallizing on the surface of fly ash particles can, nevertheless, reduce their dissolution due to reduced contact with hydroxyl ions [23]. Consistent with a study by Kim et al. [27], analcime was synthesized above 150 °C (N9_A). Kumar and Jena [28] convectively synthesized analcime at 150 °C (20 h, 3 M NaOH). The crystallization of analcime is thus conditioned by higher temperatures. However, in TS experiments with the same microwave phase, analcime was no longer formed. Although the temperature of the conventional synthesis stage of N9_B and N9_C was 180 °C, more than 98% of sodalite was prepared due to the addition of 30 mL of 30% LiCl.

The conclusion by Inada et al. [24] that the zeolitic phase does not form after 2 h of microwave irradiation of fly ash in 2 M NaOH was not confirmed. However, it is necessary to note that the temperature in the study by Inada et al. [24] was 100 °C (possibly higher), and the synthesis time in this study was extended by a 30 min increase to 120 °C. Makgabutlane et al. [25] prepared a small amount of sodalite after only 20 min of microwave irradiation (300 W). The authors determined that the crystallinity of FAZs is mainly dependent on time. When the irradiation time was reduced to 10 min and the power was increased to 900 W, no sodalite was formed. The type of microwave device greatly affects the crystallization of FAZs. While Inada et al. [24] prepared FAZs in a structurally modified microwave oven, Makgabutlane et al. [25] performed the syntheses in a Sineo UWave-1000 extraction reactor. It is necessary to mention that the total time of microwave syntheses in this study was not 150 min. At release temperatures below 80 °C and a nitrogen release rate of 10 bar∙min^−1^, the reactor was opened approx. 30 min, 40 min, and 95 min after the end of microwave heating at 120 °C, 150 °C, and 180 °C, respectively. In all microwave and TS experiments (120 °C, 150 °C), the delay was unified to 40 min. This forced crystallization phase could enhance the crystallization of FAZs. Inada et al. [24] observed an almost identical increase in the intensity of the 65 ppm peak, which is characteristic of the tetrahedral coordination of Al−O bonds, in the NMR (nuclear magnetic resonance) spectra of the products after 120 min of microwave and convection heating (compared to the spectrum of raw fly ash). However, zeolite NA-P was determined only in the convection product. Therefore, the NMR results (microwave product) could be correlated with the formation of an aluminosilicate gel. The formation of a gel, a highly reactive precursor for FAZs, is characteristic of the second phase of zeolitization [24,26]. The formation of the aluminosilicate gel, combined with the forced crystallization phase, may explain the high FAZ content in products obtained from microwave syntheses. The highest sodalite crystallinity was achieved in microwave syntheses (120 °C and 150 °C) with a Si/Al ratio of 1:2 and the use of 4 M NaOH.

#### 3.3.2. Three-Stage Syntheses

Sample No. 1 LED was used. The parameters and results of the XRD analysis of TS synthesis products are shown in Table 8.

Sodalite nucleation was supported by the addition of 30 mL of 30% LiCl in the II. Phase. Sodalite was chosen for a wide range of applications. Synthetic sodalite was used as a catalyst for the synthesis of pharmaceutical substances [46,66]. Sodalite showed higher catalytic activity and alkalinity than catalysts, such as CsNaX and KAlMCM-41 [66]. Furthermore, FAZ sodalite showed high catalytic efficiency (conversion of 95.5 wt.%) in biodiesel synthesis, specifically the transesterification of soy oil (65 °C, 2 h) [16]. Sodalite bonded with borosilicate glass was also tested as an adsorbent for salts from high-level nuclear waste [67]. It was determined that 48 h and an alkali/fly ash ratio of 10–20 mL∙g^−1^ are required for the complete convective dissolution of quartz and mullite (150 °C). At L/S 2 mL∙g^−1^, all quartz and mullite were not dissolved [68]. Increasing the volume of NaOH (from S/L 1:7.5 to 1:30) was one of the factors that using the methodology of Längauer et al. [3], increased the crystallinity of FAZs. Wałek et al. [15] determined that the convective dissolution of fly ash (104 °C) in 2–8 M NaOH is most effective at an S/L ratio of <5 g∙dm^−3^.

In this study, the S/L ratio (II. Phase) was, therefore, chosen to be lower (1:40), and the mixtures were manually stirred. Kim et al. [7] achieved a maximum Na-P1 zeolite yield of approx. 40% during 24–48 h convection syntheses (70–200 °C). The authors stated that the yield was probably reduced because the mixtures were not stirred.

Compared, for example, with metakaolin used in the synthesis of geopolymers, the design of a unified methodology for the preparation of FAZs is more complex due to the different chemical and mineralogical composition of fly ashes [65]. During the activation of fly ashes with similar contents of SiO_4_ and Al_2_O_3_ but different ratios of amorphous and crystalline components, the dissolution and subsequent nucleation of zeolites occur at different times, resulting in the formation of different FAZs [68]. Adamczyk and Białecka [29] determined that during convection heating (120 °C), the solubility of the phases decreases in the following order: glass component, quartz, and mullite. Aldahri et al. [26] determined that during the activation of the fly ash in 1M NaOH at 90 °C (for 24 h), the least soluble component of the fly ash was quartz. The time required for the dissolution of fly ash components, as well as the degree of their reactivity, differ in many studies. Some authors determined amorphous [6,8,16,24], others crystalline aluminosilicates [3], to be more reactive. All quartz and mullite were convectively dissolved after 48 h under the following conditions: 160 °C and 2 M NaOH [7] or 150 °C and 3 M KOH [36]. However, Fernández-Pereira et al. [6] did not observe an increase in FAZ yield when extending the convective heating from 24 h to 48 h (100 °C, S/L 1:5). In accordance with the mentioned studies, the initial time of convection heating in this study was chosen to be 24 h. During the II. Phase, the formation of an aluminosilicate gel was observed in PTFE bottles, as described by other authors [5,24,26].

In the T2_A product, 76.99% sodalite and 23.01% hydrosodalite were analyzed. Relative to T2_A, the sodalite content was increased to 100% by changing one of the following parameters:

- Extending the crystallization time to 7 days (T2_B);
- Prolonging the convective phase to 48 h (T2_C);
- Increasing the aluminum content (Si/Al ratio of 1:2) (T2_D);
- Increasing the temperature of the convective phase to 150 °C (T5_A).

The contents of FAZ and sodalite in the products T2_B and T2_C compared to the microwave product M2_B (the same conditions as Phase I) increased by 77% and 99.27%, respectively. It was confirmed that microwave radiation in the initial stage of synthesis increases the crystallization of FAZ, as previously reported by other authors [24,25]. Synthesis products with 4 M LiOH contained mainly an amorphous phase without FAZ (T2_E) or <1% sodalite (N9_D). Although most of the crystalline components were probably dissolved in 4 M LiOH, the solution was evaluated as unsuitable for subsequent zeolitization. It was confirmed that Na^+^ ions have a higher zeolitization ability than the smaller ones, but due to hydration, bulkier Li^+^ [65]. The effectiveness of the activator was determined to decrease in the following order: 4 M NaOH > 2 M NaOH > 4 M KOH > 4 M LiOH. The addition of 30 mL of 30% LiCl was determined to lead to exclusive sodalite crystallization in the temperature range of 120–180 °C (4 M NaOH).

#### 3.3.3. Three-Stage Syntheses under Optimal Conditions

**Scheme 3:**
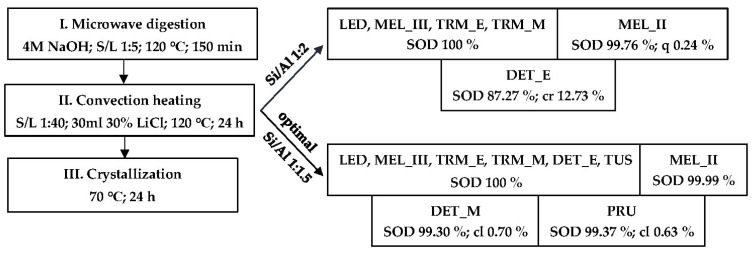
Results of XRD analysis of products depending on the Si/Al ratio and sample type.

Pure-phase sodalite was prepared with the lowest energy requirement in experiment T2_D. Under the conditions of T2_D with Si/Al ratios of 1:2 or 1:1.5, syntheses were conducted with samples 1–9 (Scheme 3).

The results of the XRD analysis (Scheme 1) show that the Si/Al ratio of 1:2 was optimal for fly ashes containing >16% aluminum. Fly ash DET_E contains 14.68% Al. In the product of sample DET_E, 12.73% of corundum was analyzed, indicating that the addition of Al_2_O_3_ (Si/Al 1:2) was excessive.

**Figure 5:**
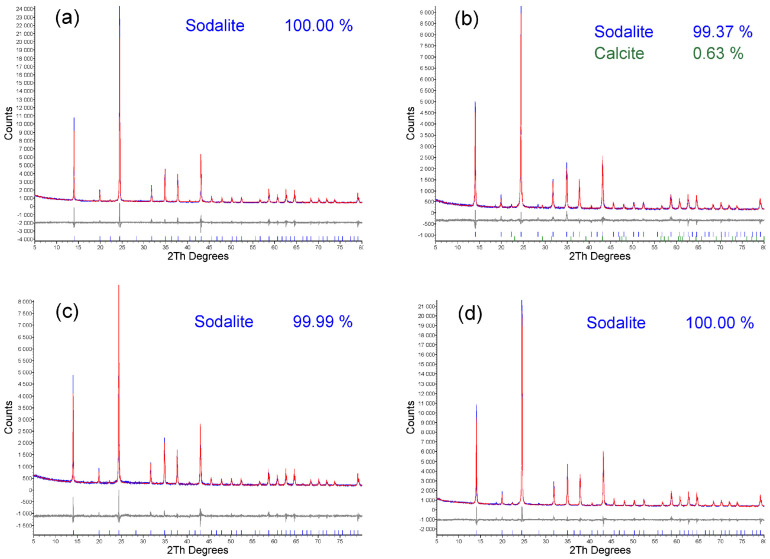

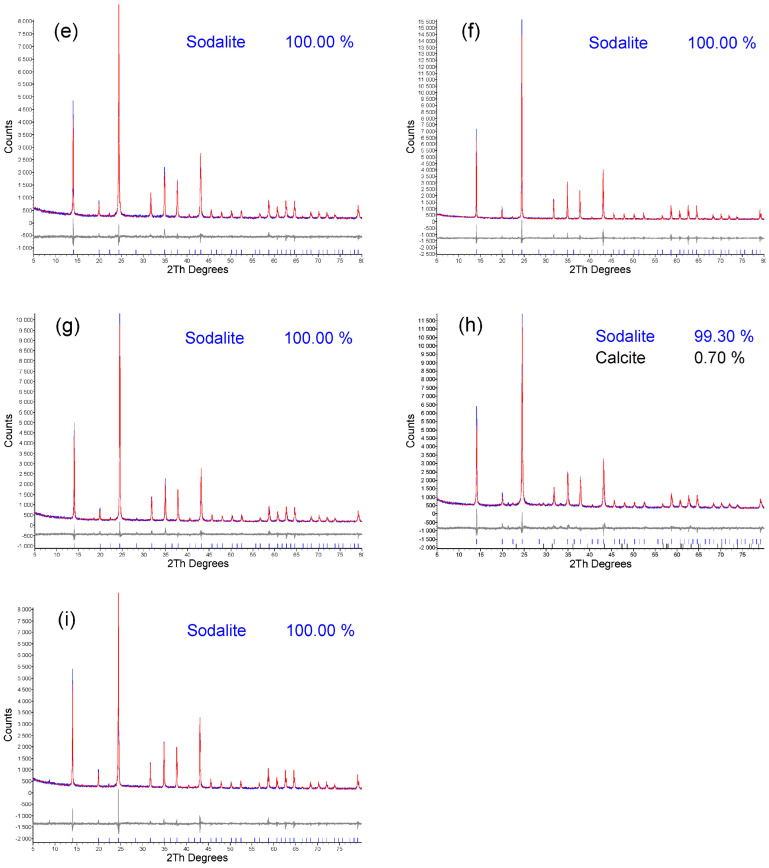
XRD patterns of the (**a**) LED; (**b**) PRU; (**c**) MEL_II; (**d**) MEL_III; (**e**) TRM_E; (**f**) TRM_M; (**g**) DET_E; (**h**) DET_M; (**i**) TUS fly ashes.

In accordance with energy minimization, during the formation of tetrahedral coordination, Si–O–Al linkages are preferentially formed over Si–O–Si. A high concentration of aluminum in the synthesis mixture accelerates the formation of an aluminosilicate framework [69]. Even the formation of Al–O–Al linkages, which contradicts Lowenstein’s rule, was predicted using density functional theory [70]. The optimal Si/Al ratio was eventually determined to be 1:1.5 (Scheme 3). Under optimal conditions, pure-phase sodalite was prepared from seven out of nine fly ashes. Only from the fly ashes DET_M and PRU was sodalite with <1% calcite synthesized (Figure 5).

The results of the XRD analysis of the products correlate with the calcium content in the raw fly ashes. DET_M and PRU fly ashes contain 1.84% and 1.44% calcium, respectively. To compare the efficiency of HT synthesis without microwave decomposition, a study by Längauer et al. [3] can be cited. Längauer et al. [3] conducted a convective synthesis (120 °C, 24 h) followed by a crystallization phase (50 °C, 16 h). The highest sodalite content (43.79%) was achieved under the following conditions: 4 M NaOH, 4 mL 10% LiCl addition, an S/L ratio of 1:30, and a Si/Al ratio of 1:1.

In experiment E183, after 183 days of activation in 4 M NaOH at room temperature, 21.38% of sodalite was prepared. The results correspond to the study by Franus [12], who prepared 42–55% of zeolite Na-X after a 12-month activation of fly ash in 3 M NaOH (S/L 1:40) at room temperature. When the amount of the sample was increased, 100% sodalite (VL_20) was prepared under optimal conditions (TS synthesis). Experiment VL_20 confirmed that microwave decomposition can be performed directly in a PTFE insert, not through an absorption medium, i.e., in reaction vessels.

**Figure 6:**
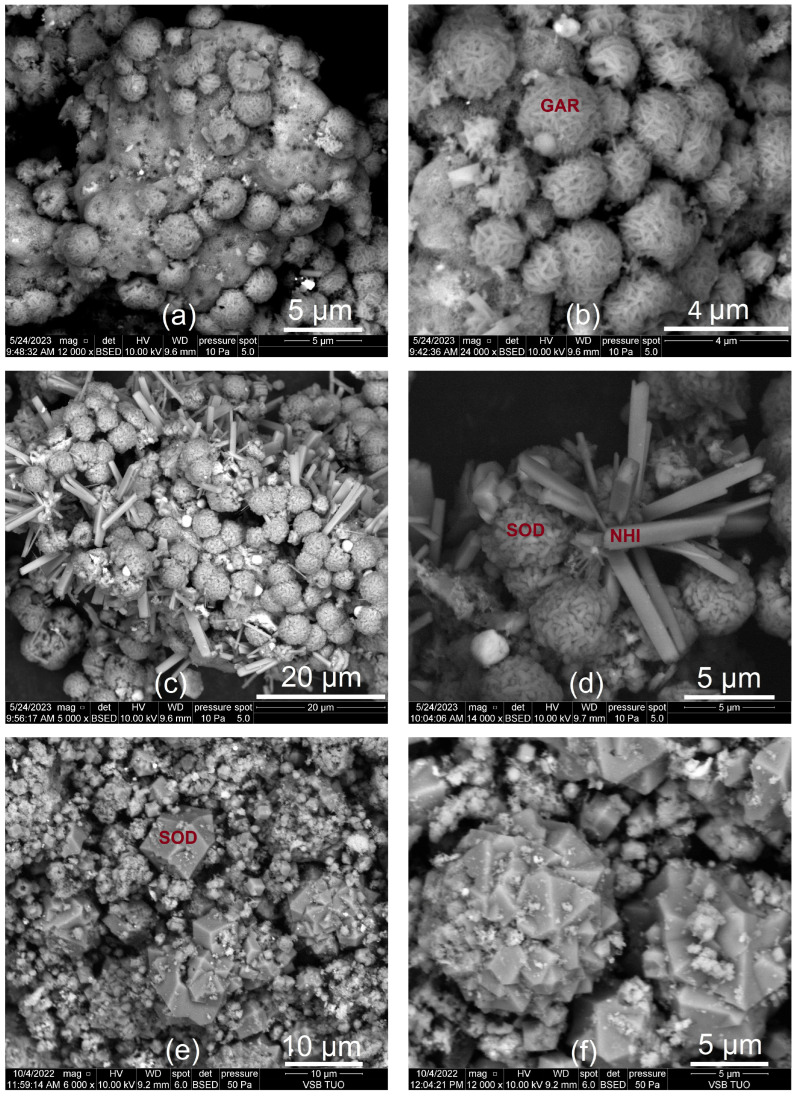
SEM images: microwave products (**a**,**b**) M2_C—garronite-Ca, (**c**,**d**) M5_C—sodalite and nepheline hydrate I; (**e**,**f**) product of three-stage synthesis under optimal conditions (sample LED)—sodalite.

The confirmation of XRD results was conducted using SEM (Figure 6).

**Figure 7:**
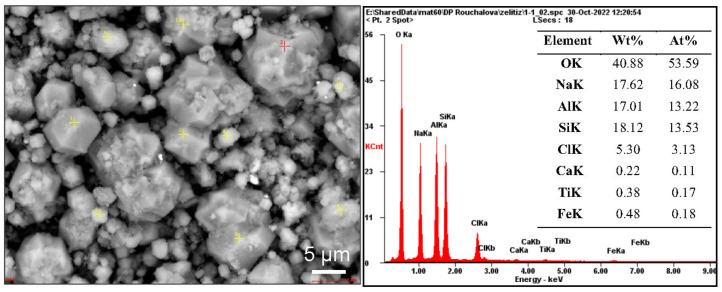
SEM image of one L_20 product with ED XRF spot analysis (red cross).

The synthesis conditions of the microwave product M2_C shown in Figure 6a,b are equivalent to the conditions of the I. Phase of TS syntheses under optimal conditions. In the product M2_C (120 °C), garronite-Ca crystallized on the surface of fly ash residues was identified. Crystals of FAZ on the surface of fly ash residues have been observed in several studies [5,8,26,27,30,36,40,41]. In the product M5_C (150 °C), intricate growths of sodalite tetrahedra and NHI crystals in the shape of rhombic prisms can be seen (Figure 6c,d). The product of TS synthesis under optimal conditions contains rhombododecahedral sodalite crystals (Figure 6e,f). Synthetic sodalite is purely chloride; it is not hydroxysodalite, i.e., Na_8_[Al_6_Si_6_O_24_](OH)_2_·2H_2_O [69], nor hydrosodalite, as confirmed by the results of SEM-ED XRF analysis (Figure 7).

## 4. Conclusions

This study presents a three-stage methodology for the synthesis of pure-phase sodalite from fly ash. Under optimal conditions, the products of nine high-temperature fly ashes contained >99 wt.% sodalite. Therefore, sodalite can be used directly, and it is not necessary to separate it from fly ash residues. The methodology is effective for fly ashes (brown coal and bituminous) of different chemical and mineralogical compositions in the range of phase contents, such as quartz (4.93–34.98%) and mullite (10.91–43.23%), and the amorphous phase (50.54 –81.76%). Although it was previously determined that effective ash dissolution is conditioned by activation at 125–200 °C [68], the temperature in any of the phases did not exceed 120 °C. Pure-phase sodalite was prepared directly in suspension, not from extracts separated after alkaline mineralization. Therefore, no solid waste phase from the extract preparation is produced. The main disadvantage is the production of waste filtrate after the separation of the solid zeolitic phase. However, it has been shown that filtrates can be reused for FAZ synthesis while maintaining the CEC values of the products [6].

Achieving high yields of sodalite is the result of many factors:

- Microwave digestion was performed using SRC’s patented technology in the initial stage of synthesis in the UltraCLAVE IV autoclave;
- The effect of pressure (30 bar), which increased during microwave digestion;
- A combination of two types of heat transfer: transmission, high efficiency for dissolving quartz and mullite, and radiation, high efficiency for dissolving the glass phase;
- Optimal stirring intensity: very intense (70%) during microwave dissolution and mild manual during convection heating;
- Optimal S/L ratios: higher than 1:5 in the I. Phase, which allowed intensive absorption of microwave radiation, and lower (S/L 1:40) in the II. Phase, in which the aluminosilicate gel was formed;
- The addition of 30 mL of 30% LiCl.

This study confirmed that high-temperature fly ash is a suitable matrix for the HT synthesis of a specific type of zeolite.

## Figures and Tables

**Table 1:** Summary and technological parameters of the samples.

Sample No.	Fly Ash	Producer	YP ^3^	Coal Rank	Particle Separator
1	LED	Ledvice PP ^1^	2020	Brown coal	Electrostatic
2	MEL_II	Mělník II PP	2020	Brown coal	Mechanical + Electrostatic
3	MEL_III	Mělník III PP	2020	Brown coal	Mechanical + Electrostatic
4	TRM_E	Trmice HP ^2^	2020	Brown coal	Electrostatic
5	TRM_M	Trmice HP	2020	Brown coal	Mechanical
6	DET_E	Dětmarovice PP	2020	Bituminous coal	Electrostatic
7	DET_M	Dětmarovice PP	2020	Bituminous coal	Mechanical
8	PRU	Prunéřov PP	2020	Brown coal	Electrostatic
9	TUS	Tušimice PP	2021	Brown coal	Electrostatic

^1^ PP: power plant; ^2^ HT: heating plant; ^3^ YP: year of production.

**Table 2:** Weight percentages of major elements in fly ashes determined by WD XRF.

Fly Ash	Major Elements (wt.%)										
	Si	Al	Fe	Mn	Ti	Na	K	Mg	Ca	P	S
LED	24.48	18.47	4.47	0.02	1.96	0.21	0.51	0.43	0.95	0.06	0.07
MEL_II	21.36	17.90	6.22	0.04	4.25	0.24	0.66	0.39	1.11	0.15	0.15
MEL_III	23.53	16.38	5.87	0.04	2.96	0.21	0.91	0.44	1.17	0.12	0.15
TRM_E	25.19	17.32	5.18	0.03	1.83	0.14	1.03	0.46	0.92	0.08	0.07
TRM_M	24.52	16.17	4.20	0.02	1.27	0.12	1.06	0.42	0.68	0.07	0.13
DET_E	23.57	14.68	4.37	0.06	0.77	0.56	3.24	1.16	2.33	0.24	0.36
DET_M	30.05	11.15	3.68	0.06	0.57	0.32	2.63	0.86	1.84	0.06	0.08
PRU	24.45	16.12	6.06	0.05	1.02	0.29	1.31	0.60	1.44	0.09	0.20
TUS	25.17	14.99	8.61	0.03	0.74	0.29	1.31	0.56	1.21	0.07	0.16

**Table 3:** Concentration of minor elements in fly ashes determined by WD XRF.

Fly Ash	Minor Elements (mg∙kg^−1^)														
	Rb	Cs	Sr	Ba	La	Ce	Zr	V	Nb	Cr	Ni	Cu	Zn	Pb	As
LED	66	33	304	511	109	238	581	384	120	286	122	139	123	24	15
MEL_II	111	63	549	883	153	321	606	469	166	204	99	208	143	49	69
MEL_III	75	71	653	1039	199	395	796	588	230	159	95	269	152	53	102
TRM_E	115	39	385	525	113	258	486	332	98	208	111	108	137	35	19
TRM_M	126	40	353	630	98	221	354	255	73	190	95	95	98	30	15
DET_E	173	–	453	1777	46	87	212	147	25	122	109	153	681	198	28
DET_M	114	–	196	678	30	55	196	74	17	95	63	55	71	65	–
PRU	140	–	289	690	62	153	244	204	40	128	124	127	195	84	38
TUS	167	42	352	719	92	217	213	278	33	196	126	125	331	90	75

**Table 4:** Results of fly ash determinations (the F-AAS, LOI, and pH of leachates).

Fly Ash	Content Al ± RSD ^1^ (%)	Content Fe ± RSD (%)	LOI (%) Average ^2^ ± SD ^3^	pH
LED	11.2 ± 3.0	3.1 ± 0.3	1.05 ± 0.11	7.4
MEL_II	13.8 ± 1.0	4.5 ± 0.1	1.52 ± 0.15	6.6
MEL_III	14.8 ± 0.5	4.7 ± 0.2	1.99 ± 0.17	6.3
TRM_E	9.3 ± 0.9	3.9 ± 0.3	1.33 ± 0.20	9.8
TRM_M	14.8 ± 4.2	2.8 ± 0.1	6.86 ± 0.81	8.6
DET_E	15.1 ± 4.4	5.1 ± 0.6	2.88 ± 0.74	10.2
DET_M	10.2 ± 1.7	4.0 ± 0.4	0.61 ± 0.39	10.6
PRU	16.4 ± 3.0	7.4 ± 0.5	1.37 ± 0.82	6.3
TUS	12.6 ± 1.5	6.7 ± 0.7	0.92 ± 0.01	6.1

^1^ Relative standard deviation; ^2^ arithmetic average from a set of three data, the set having no outliers; ^3^ standard deviation.

**Table 5:** Results of XRD analysis of fly ashes.

Fly Ash	Phase (wt.%)						
	Quartz	Mullite	Cristobalite Low	Sanidine	Magnetite	Anhydrite	Amorphous
LED	4.93	43.23	–	–	1.11	–	50.72
MEL_II	9.22	34.95	–	1.98	1.56	1.77	50.54
MEL_III	10.38	31.48	–	–	1.92	0.68	55.55
TRM_E	5.67	36.28	3.88		3.30		50.87
TRM_M	13.23	18.46	–	–	1.04	1.01	66.27
DET_E	5.85	11.60	–	–	0.62	0.16	81.76
DET_M	34.98	10.91	–	–	1.00	–	53.11
PRU	8.42	27.34	–	–	2.91	–	61.32
TUS	6.72	26.50			2.13		64.66

**Table 6:** Iron contents in fly ashes and magnetic and non-magnetic fractions, and the results of magnetic separation indicators (yields, recoveries, and efficiencies).

Fly Ash	Indicator (%)										
	*a*	*b*	*c*	*v_c_*	*v_b_*	*m_c_*	*m_b_*	*w_c_*	*w_b_*	*η_c_*	*η_b_*
LED	4.47	2.21	41.35	5.77	94.23	53.41	46.59	3.54	96.46	49.87	−49.87
MEL_II	6.22	2.94	47.35	7.39	92.61	56.22	43.78	4.15	95.85	52.08	−52.08
MEL_III	5.87	2.70	48.06	6.99	93.01	57.22	42.78	3.86	96.14	53.36	−53.36
TRM_E	5.18	2.46	46.17	6.22	93.78	55.46	44.54	3.53	96.47	51.93	−51.93
TRM_M	4.20	2.01	36.32	6.38	93.62	55.20	44.80	4.24	95.76	50.95	−50.95
DET_E	4.37	2.08	44.9	5.35	94.65	54.95	45.05	3.08	96.92	51.87	−51.87
DET_M	3.68	1.67	37.9	5.55	94.45	57.14	42.86	3.58	96.42	53.56	−53.56
PRU	6.06	2.86	47.9	7.10	92.90	56.16	43.84	3.94	96.06	52.22	−52.22
TUS	8.61	3.78	49.1	10.66	89.34	60.78	39.22	5.94	94.06	54.84	−54.84

**Table 7:** Designation and characterization of crystalline phases analyzed in HT synthesis products by XRD.

Identification	Phase	Code ^1^	Empirical Formula
SOD	Sodalite	SOD	|Na_8_ Cl_2_|[Al_6_Si_6_O_24_] [58]
HYD	Hydrosodalite	SOD	Na_6_[AlSiO_4_]_6_(H_2_O)_8_ [59]
GAR	Garronite-Ca	GIS	NaCa_2,5_(Si_10_Al_6_)O_32_·13H_2_O [60]
NHI	Nepheline hydrate I	JBW	Na_3_(AlSiO_4_)3,1–2H_2_O [61]
MER	Merlinoite	MER	|K_5_Ca_2_ (H_2_O)_24_|[Al_9_Si_23_O_64_] [58]
ANA	Analcime	ANA	Na(Si_2_Al)O_6_·H_2_O [60]
q	Quartz	–	SiO_2_
s	Sylvite	–	KCl
cl	Calcite	–	CaCO₃
cr	Corundum	–	Al₂O₃

^1^ Framework type code according to the International Zeolite Association (IZA).

**Table 8:** FAZs determined by XRD in TS synthesis products depending on the parameters.

Identification	FAZs	I. Microwave Digestion				II. Convection Heating		III. Crystallisation	
		Si/Al	Activator	T_M_ (°C)	t_M_ (min)	T_S_ (°C)	t_S_ (h)	T_C_ (°C)	t_c_ (Day)
T2_A	SOD 76.99%; HYD 23.01%	1:1	4 M NaOH	120	150	120	24	70	1
T2_B	SOD 100%	1:1	4 M NaOH	120	150	120	24	70	7
T2_C	SOD 100%	1:1	4 M NaOH	120	150	120	48	70	1
T2_D	SOD 100%	1:2	4 M NaOH	120	150	120	24	70	1
T2_E	–	1:1	4 M LiOH	120	150	120	24	70	7
T5_A	SOD 100%	1:1	4 M NaOH	150	150	150	24	70	1
T5_C	SOD 99.96%; s 0.04%	1:1	4 M NaOH	150	150	150	48	70	1
N9_B	SOD 100%	1:1.5	4 M NaOH	180	150	180	24	70	1
N9_C	SOD 98.95%; cl 1.05%	1:1.5	4 M NaOH	180	150	180	24	50	1
N9_D	SOD < 1%	1:1	4 M LiOH	180	150	180	24	70	1

## Data Availability

Data are contained within the article.

## Abbreviations

ED XRF	Energy-dispersive X-ray fluorescence
F-AAS	Flame atomic absorption spectrometry
FAZs	Fly ash zeolites
HP	Heating plant
HT	Hydrothermal
IZA	International Zeolite Association
LOI	Loss on ignition
NHI	Nepheline hydrate I
NMR	Nuclear magnetic resonance
PP	Power plant
PSD	Particle size distribution
RSD	Relative standard deviation
SD	Standard deviation
SEM	Scanning electron microscope
S/L	Solid-to-liquid ratio
SRC	Single Reaction Chamber
TS	Three stage
XRD	X-ray diffraction
WD XRF	Wavelength-dispersive X-ray fluorescence
